# Prediction of Mineral Composition in Wheat Flours Fortified with Lentil Flour Using NIR Technology

**DOI:** 10.3390/s23031491

**Published:** 2023-01-29

**Authors:** Iván Martínez-Martín, Miriam Hernández-Jiménez, Isabel Revilla, Ana M. Vivar-Quintana

**Affiliations:** Food Technology, Polytechnic High School of Zamora, Universidad de Salamanca, Avenida Requejo 33, 49022 Zamora, Spain

**Keywords:** NIR, lentil flour, minerals, fortified flour

## Abstract

Lentil flour is an important source of minerals, including iron, so its use in food fortification programs is becoming increasingly important. In this study, the potential of near infrared technology to discriminate the presence of lentil flour in fortified wheat flours and the quantification of their mineral composition is evaluated. Three varieties of lentils (Castellana, Pardina and Guareña) were used to produce flours, and a total of 153 samples of wheat flours fortified with them have been analyzed. The results show that it is possible to discriminate fortified flours with 100% efficiency according to their lentil flour content and to discriminate them according to the variety of lentil flour used. Regarding their mineral composition, the models developed have shown that it is possible to predict the Ca, Mg, Fe, K and P content in fortified flours using near infrared spectroscopy. Moreover, these models can be applied to unknown samples with results comparable to ICP-MS determination of these minerals.

## 1. Introduction

Millions of tons of flour from different sources are consumed by people around the world [1]. In this context, vegan and vegetarian diets have boosted the development and consumption of new plant sources of protein, minerals, vitamins and bioactive compounds in which legume flour has become very important [2,3,4]. In addition, food intolerances have promoted the development of a wide variety of plant-based flours and the development of derived products [5,6]. Flours from chickpea, amaranth, quinoa, lentils and mushroom [7,8] as well as flours from different cereals and pseudocereals [6] are being widely used alone and/or combined with wheat flour for the development of new products [9].

Flours provide different micronutrients in the diet (vitamins, minerals or other nutrients) through the process of fortification in which nutrients or non-nutrient bioactive components are added to edible products. Owing to their low cost, high mineral content fortified flours have been used in many public health strategies in underdeveloped countries to combat malnutrition and diseases caused by vitamin or mineral deficiencies [9,10]. However, in developed countries, fortification is mainly used to meet the demand for health-promoting products with high nutritional value [11,12]. Lentil flour is a good alternative to be used as a fortifier alongside wheat flour to improve its nutritional quality. This flour is rich in bioactive compounds and phytochemicals [13], low in fat [14], high in fiber [15], antioxidant properties [16] and minerals [17]. The mineral elements of lentils include Fe, Zn, Cu, Mn, Mo, Mg, P, Ca and K [3].

Preparations containing flour mixes intended for the processing industry and even for direct sale to the consumer have increased their presence in recent years. However, these products are among the foods with the highest incidence of adulteration [7,18,19]. Both industry and consumers are increasingly concerned about the safety, quality and authenticity of the products for sale [20]. Food control techniques are therefore promoted by consumers, regulatory bodies and the food industry to detect fraud in labeling [7].

In this context, several techniques have been evaluated, including authentication based on scanning electron microscopy (SEM) [21], molecular biology techniques (PCR) [22], fluorescent electron spectroscopy [23], imaging spectroscopy methods [24], nuclear magnetic resonance (NMR) spectroscopy methods [25] and gas chromatography (GC) or gas-mass chromatography (GC-MS) separation techniques [26]. Near infrared spectroscopy (NIR) has been extensively developed in the cereal industry for the determination of physico-chemical parameters, technological quality and geographical origin in grains and seeds of wheat, barley, rye, sorghum, oats and legumes [27,28,29,30,31,32,33] and in barley, wheat and lentil flours [34,35,36,37]. Salgó y Gergely [32] studied wheat seed maturation processes by analyzing changes in moisture, carbohydrate and protein contents. Armstrong et al. [38] analyzed protein, moisture and hardness index contents of whole grain wheat. Within legumes, NIRS has been applied to predict protein content in pea seeds and flours [39] and proximate composition in soybean [40] and faba bean [41]. Other parameters such as phytochemical composition, antioxidant activity or phenolic composition [42] and anti-nutritional factors [43] have also been predicted using NIR in common beans. In the case of lentils, the differentiation of geographical origin [29,44], the nutritional quality [45] and the protein and amino acid content [46] have also been assessed by NIRS. In relation to wheat flour, NIR technology has been applied to assess the chemical composition (moisture, protein, ash and some functional substances of wheat flour) [47], as well as amylose contents [38]. In addition, NIR spectroscopy has also been applied to detect the presence of chemical additives [48] and biological contaminants in wheat flour [49]. The applicability of NIR technology to the analysis of cereals and legumes is based on the characteristic absorption peaks in the near-infrared region of the hydrogen-containing groups of each component of these products [50].

NIR technology has also been applied to the detection of wheat flour fraud [50,51]. Thus, the work carried out by de Almeida et al. [52] using NIR spectroscopy and coupled digital imaging obtained excellent results in determining the cassava starch content incorporated into wheat flour used for bread making. Similarly, Tao et al. [53] were able to distinguish pure wheat flour from wheat flour adulterated with cassava flour using a portable micro-NIR spectrometer. Adulteration with cassava and maize flours has also been studied in organic wheat flours [54], and the presence of peanut flour or walnut flour in the adulteration of whole wheat flour has been evaluated using the NIR-HSI technique [55,56]. Other adulterations, such as the presence of taro and sago flours [57], cheap grain [24], azodicarbonamide [58] and the adulteration of durum wheat flour with common wheat of bread-making quality [59], have also been studied using NIR technology.

The aim of this study is to evaluate the viability of NIR technology in the analysis of wheat flour fortified with lentil flour in order to assess that wheat flours fortified with lentil flour fulfil the declared composition. To this purpose, the study aims to predict the percentage of lentil flour and the final mineral content (Ca, P, K, Fe, Mg) of the fortified flours from the NIR recording obtained from these flours.

## 2. Materials and Methods

### 2.1. Samples

A total of 153 flour samples were analyzed as described in Table 1. Firstly, 3 commercial wheat flours were purchased. The wheat flour used was 75% extraction with a strength of 200 · 10^−4^ Joules, belonging to the guaranteed brand “Harina Tradicional Zamorana”. Subsequently, 60 lentil flours were manufactured. Lentil flours were obtained by grinding whole lentils in a micro-hammer mill (Culatti Type MFC, Germany) and sieving them to a final particle size of less than 0.140 mm. Three different varieties of brown lentils grown in Spain (Lens culinaris var. Castellana, var. Guareña and var. Pardina) were used to obtain the flours. The lentils were supplied by the Centro de Legumbres (Salamanca, Spain) and harvested from 10 different plots in the regions of Zamora and Salamanca (Spain) in order to achieve as much variability in their mineral content as possible. Finally, 90 fortified flours were prepared by mixing wheat flour with different amounts of lentil flour. In all cases, 50 g of flour were prepared. The wheat flours and lentil flours used in the mixture were chosen randomly, taking care that for each level of fortification 10 mixtures were prepared with each lentil variety. The fortified flours were prepared by mixing wheat flour with 25, 50 and 75% of the different lentil flours.

### 2.2. Mineral Content

Mineral concentrations were determined by ICP-MS (Mg, P, K, Ca, Fe). Samples (0.2 g) were introduced in Teflon vessels with HNO_3_ and a Milestone digestion microwave system was used. Concentrations were determined using an Agilent 7800 ICP mass spectrometer (Agilent, Santa Clara, CA, USA) with the following operating conditions: Reference Power 1550 W, Plasma Air flow 15 L/min, Auxiliary Air flow 0.9 L/min and Nebuliser Air flow 0.99 L/min). The quantification was carried out by using certified standard solutions (Scharlab, Spain), and the results were expressed in mg/kg of flour.

### 2.3. NIR Spectroscopy and Chemometric Methods

**Figure 1:**
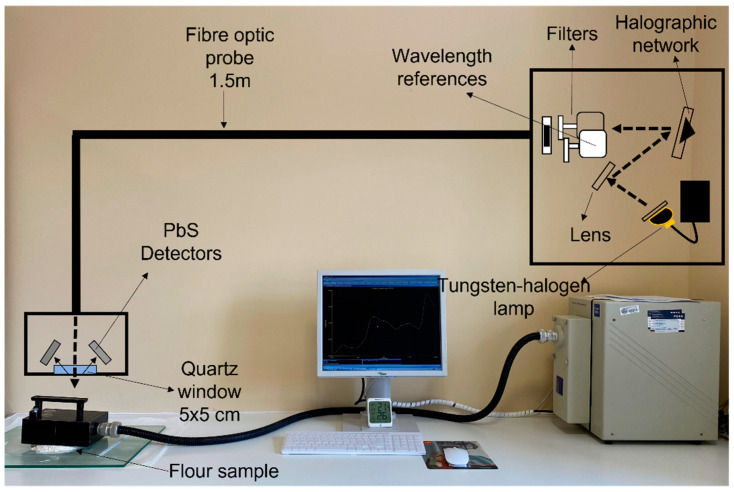
Methodology used for recording the spectra of flour and fortified flour samples.

The flours were analyzed with a Foss NIR System 5000 monochromator (Foss NIRSystems, Silver Spring, MD, USA). A 1.5 m 210/210 remote reflectance fiber probe (Ref. No. R6539-A) was used with a 5 × 5 quartz window area. The spectral range used was between 1100–2000 nm at 2 nm intervals. The measurement was carried out by direct application of the fiber-optic probe to 10 g of flour, as shown in Figure 1. The diffuse reflectance signal of the NIR spectrum is represented as log(1/R) (R = reflectance). The chemometric treatment of the spectral data was carried out with WinISI 4.10 software (Infrasoft International, State College, PA, USA).

Firstly, a principal component analysis (PCA) was performed to detect spectral outliers using the Mahalanobis distance as a criterion for the validity of the samples [60], considering the overall outlier H ≥ 3. The spectra obtained from the flour samples, which met the above criteria, were randomly divided into two groups known at the calibration set with 80% of the total samples (120 samples) and the validation set with 20% of the total samples (33 samples).

Calibration was then carried out using the spectra of the samples and their respective mineral content data analyzed in the laboratory as well as the percentage of lentil flour present in the fortified samples. Four different spectral treatments were applied to correct the possible scattering effects of the samples: multiplicative scatter correction (MSC), standard normal variant (SNV), DeTrend (DT) and SNV-DT. In addition, four different derivative math treatments were used. The calibration equations were generated using the modified partial least squares (MPLS) regression method. The equation generated was developed by cross-validation with the selection of an optimal number of factors to avoid over-fitting. To evaluate the developed models, the performance deviation (RPD) and the cross-validation mean square error (SECV) were analyzed.

The selection of the best calibration equation was made based on the coefficient of determination R^2 and the RMSEC (root mean square error of calibration). Finally, the equations obtained were applied to the validation set which was not used for calibration (33 samples). The values predicted from the equations generated were compared with the mineral content data determined in the laboratory using the Student’s *t*-test for paired values.

Discrimination of the flours according to the percentage of lentil flour was carried out using the Residual Mean Squares (RMS-X) supervised pattern recognition method. The same spectral pre-treatments as described above were tested in this case. Some 80% of the samples were used to generate the model, and the remaining 20% were used for subsequent validation. The discrimination models obtained were evaluated according to the percentage of well-classified samples during both calibration and external validation. The sensitivity of the models was calculated according to the expression proposed by [61].

## 3. Results

### 3.1. Spectra Collection 

**Figure 2:**
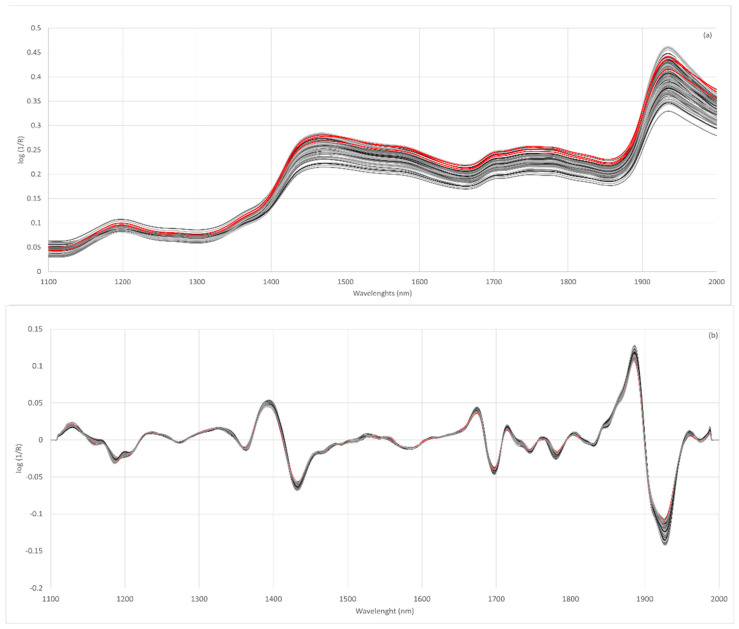
Raw NIR spectra of all flour samples analyzed (**a**), spectra after second derivative SNV 2,4,4,1 (**b**); wheat flours (red line), lentil flours (dark grey line) and fortified flours (light grey).

Figure 2a shows the spectra obtained for wheat, lentil and fortified flours. The spectra of all the flours analyzed show a similar spectral pattern. The main absorption bands are located at 1464–1470 nm and 1930–1940 nm. These bands have already been described in the absorption spectra of rice flours [62]. The 1466 nm band was related to the O-H (starch) and N-H (protein) stretch first overtone [63] and the 1934 nm band to the O-H bend second overtone related to water [62].

The raw spectra of the flours show a large vertical shift with respect to the baseline, which affects the whole spectral region analyzed. These differences could be related to the scattering behavior of samples with different particle sizes. Scattering is the main source of spectral variability when analyzing milled products such as flour although the use of pre-treatment for the original spectra allows for the elimination of particle size effects [47,64]. Considering the above, it is necessary to evaluate different spectral pre-treatments [65]. The effects of scattering were removed using multiplicative scatter correction (MSC), standard normal variate (SNV), detrend (DT) or SNV-DT and combinations of these. Furthermore, different mathematical treatments were tested in the spectra obtained.

The Norris–Williams spectral derivative was used where the first digit was the number of the derivative, the second was the interval during which the derivative was calculated, the third was the number of data points in an average or smoothing, and the fourth was the second smoothing [66]. For most of the treatments tested, it can be observed that the discrimination capacity is lower when using the treatment identified as 0,0,1,1. The results showed that the pre-treatments of the second derivative were those most efficient in preventing this dispersion (Figure 2b).

### 3.2. Discrimination of the Percentage of Lentil Flour in Fortified Flour

In an initial stage, the ability of discriminant models to classify samples attending to the different percentages of the lentil flour contained in the mixtures was evaluated. For this purpose, the Residual Mean Squares method was used. Table 2 shows the results obtained in the discrimination of flour blends for different pre-treatments. The results are highly satisfactory; in all cases, the discrimination capacity of the samples is higher than 90% both in the samples used for calibration and in those reserved for validation. These results show that even when the spectra are not treated with spectral pre-treatments (identified as none), it is possible to correctly classify 100% of the flours in one of the four groups established for the presence of lentil flour. However, the classification improves when mathematical pre-treatments are applied to the raw spectra. The results of sensitivity obtained (Table 2) show that misclassification errors only occurred in samples of groups of 100 or 75. The sensitivity informs us of the proportion of samples belonging to a group that has been correctly classified.

Furthermore, the possibility of discriminating the fortified samples according to the variety of lentil used in the production of the flour was tested (Table 2). Although brown varieties with yellow cotyledons were used in all cases, the NIR technology can discriminate the lentil variety in a highly efficient manner. In this case, the discrimination capacity reached 100% in the samples used for calibration and 96.67% for the data used for validation. As can be seen in Table 2, the sensitivity obtained differs according to the lentil variety. Lentils of the Guareña and Castellana varieties show lower sensitivity. Both varieties belong to the species *Lens culinaris* subsp. *Macrosperma*; however, the Pardina variety belongs to subsp. *microsperma* [67], which could explain the results obtained.

NIR technology has already been applied in the discrimination of flours with correct classification values above 90% in sweet potato flour and its adulterations [68] and in the discrimination of wheat flour samples based on geographical origin [69]. In addition, it has shown a good discrimination capacity when applied to noodle flour [70] and an accuracy of between 92.8 and 100% for wheat flour to discriminate between spelt flour and einkorn flour [71].

### 3.3. Mineral Composition of Lentil Flours

Table 3 shows the average mineral contents (potassium, phosphorus, magnesium, calcium and iron) of the flours used in this study. In relation to the lentil flours, it can be observed that for the three brown varieties analyzed, potassium is the macro-element found in the highest concentration in agreement with the results shown by Faris et al. [3]. The average values found for this element were 0.87, 0.94 and 0.81 g/100 g for the varieties Castellana, Guareña and Pardina, respectively. In relation to phosphorus, the concentrations found were between 0.27 and 0.45 g/100 g, with a significantly lower concentration (*p* < 0.05) in the flours of the Castellana variety. The magnesium contents did not show statistically significant differences between the different varieties (*p* ≤ 0.05). However, the Castellana variety shows a statistically (*p* ≤ 0.05) higher calcium content than the Guareña and Pardina varieties. The concentrations found for all minerals in the present study agree with those described for green, yellow and red lentil flours [17,72].

Lentils have traditionally been considered a good source of iron [17,73]. Their high bioavailability makes lentils an important dietary source of this mineral [74]. In the lentils analyzed in this study, the iron concentrations found ranged from 42.30 to 115.00 mg/kg. There are no statistically significant differences (*p* < 0.05) between the three lentil varieties owing to the large variability found between the different growing areas from which the lentils were collected. As reported, the micronutrient content of lentils appears to vary greatly according to their location [75], the environmental conditions [76] and the genotype, with location being the most important factor in the case of iron content [77].

The results obtained show that the concentrations of all the minerals analyzed are significantly higher (*p* < 0.05) in lentil flours than in wheat flours.

Based on this mineral composition, lentil flours can be used in the fortification of wheat flours to increase their mineral content. Micronutrient malnutrition is a serious problem, especially in developing countries [78]. As reported by Bouhlal et al. [79], the fortification of wheat flour with lentil flour is a good option for controlling iron deficiency as well as protein malnutrition and diabetic disease, while the functional properties of these blends are suitable for use in the food industry.

The preparing of mixtures by incorporating different amounts of the various lentil flours into three different wheat flours resulted in flours with increasing concentrations of phosphorus, potassium and iron, as expected. All these flours provide us with a sample set with a wide enough range of mineral concentrations to develop a suitable calibration model.

### 3.4. Determination of Mineral Composition by NIRS

#### 3.4.1. NIR Calibration Equation of Mineral Content

For the development of the calibration, a principal component analysis (PCA) was first performed. The spectral variability explained was above 99% in all cases, and between 6 and 10 principal components were required for each mineral analyzed. Calibrations were performed by modified partial least squares (MPLS) regression for each of the minerals after removing those samples that did not satisfy the spectral criteria of Mahalanobis distance (H distance). Spectra with H values equal to or greater than three were therefore discarded.

Some 120 samples were used to develop the calibration models. The best performing mathematical treatment and the calibration parameters for all minerals analyzed (Ca, Mg, K, P and Fe) are shown in Table 4. The statistical parameters (RSQ, SEC and SECV) of the calibration generated by the MPLS models were used to determine the best calibration equations (Table 4). The calibration models obtained for all the minerals analyzed were highly satisfactory. For Mg, P, K and Ca, the RSQ values obtained were higher than 0.94. Only Fe had a value lower than 0.9 (RSQ 0.87). Taking into account SEC (standard error of calibration) and SECV (standard error of cross-validation) errors, the results obtained are excellent for Mg, P, K and Ca and satisfactory for Fe. NIR spectroscopy can predict inorganic materials because mineral matter is associated with organic compounds so that the distortion of the spectrum produced may be detectable at certain wavelengths [80,81]. Minerals that do not have absorption in the near infrared region (e.g., Ca and P) can be detected indirectly through their binding to organic complexes, chelates or pigments [82]. Minerals establish a strong relationship with O-H bonds and combined C-H bonds, which allows NIR technology to be used in the quantification of minerals in foods [80]. However, minerals may exist in different forms, some of which are easier to detect with NIRS than others [81].

The calibration equations developed take into account all the wavelengths recorded when obtaining the NIR spectrum. However, not all of them contribute to the equation in the same way. Each wavelength is multiplied by a coefficient (β coefficient), the higher the value of β, the greater the contribution of that wavelength to the predictive ability of the equation developed. The results obtained in this study show that the lengths between 1212 and 1282 nm have high β coefficients in the equations of all the minerals analyzed. High coefficients are also observed at 1578 nm for Ca and Fe. In the case of K, high β coefficients are observed between 1114 and 1184 nm.

#### 3.4.2. Internal and External Validation

From the equations obtained, an internal validation was carried out through a cross-validation of the samples used in the development of the equations. To evaluate the performance of the models, the RSQ (R squared), the RPD (Relative Percent Deviation) and the SEP (standard error of prediction) values were used. The RSQ coefficient measures the correlation between the content in each mineral predicted by the model and the value obtained in the laboratory. The RPD value is the ratio of the yield to the deviation. The SEP provides an indication of the precision of our results. In the internal validation process, samples with high residual values were eliminated using the criterion T > 2.5.

**Figure 3:**
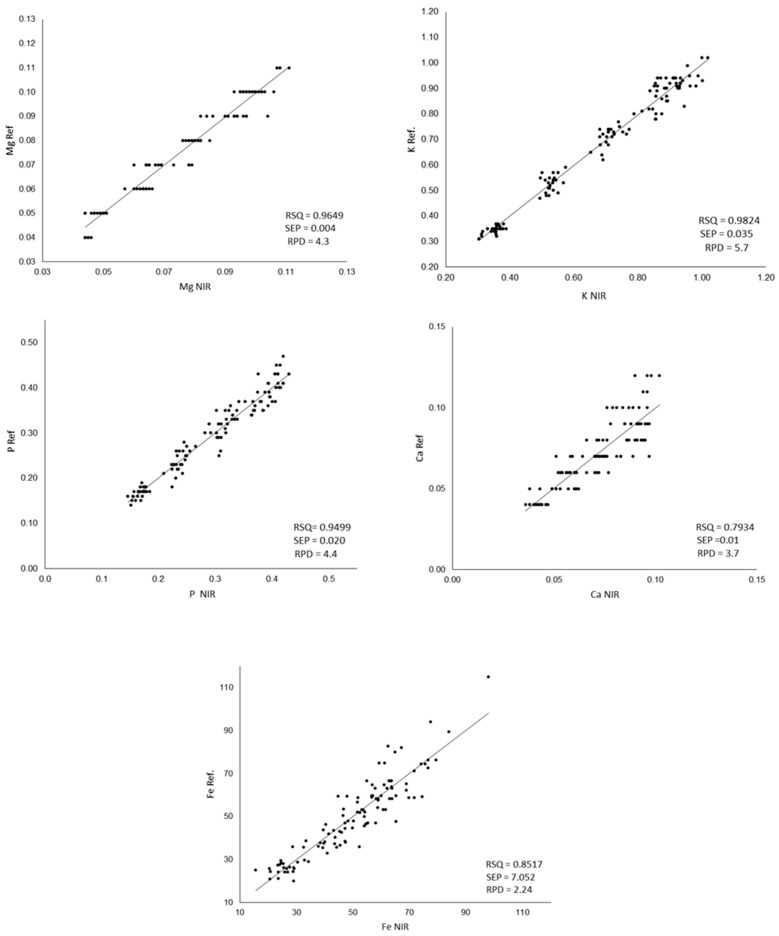
Comparison between the values measured by ICP-MS (Ref.) and the values predicted by the developed equations (NIR); RPD: ratio performance deviation; SEP: standard error of prediction, RPD: relative percent deviation. The Ca, Mg, K and P content was expressed as g/100 g of flour, and Fe content was expressed as mg/Kg of flour.

The results of the statistical descriptors of the internal validation and the correlations between the mineral content measured in the laboratory and that predicted by NIR are shown in Figure 3.

RSQ values are highly satisfactory for P, Mg and K. For Fe, a lower value is observed, as was previously shown when developing the calibration equation. Poor calibrations for Fe have been previously reported for legumes [30,83]. For Ca, the comparison between the values obtained in the laboratory and those estimated from NIR shows the lowest RSQ values. Similar results were obtained by Zhang et al. [50] in wheat flour, obtaining RSQ values of 0.7907 for Ca while obtaining RSQ of 0.9777 for P and K. The Ca does not have absorption in the near infrared region but is detected indirectly through its binding with organic complexes, chelates and pigments [82], which could explain the greater difficulty in its calibration. In relation to the standard error of prediction, only iron shows a fairly high SEP (7.052). According to the RPD values, a model is considered good when its value is >2.0, a model is considered to be in need of improvement when the values are between 1.4 and 2.0, and a model is considered to be unreliable if its value is <1.4 [84].

Taking these values into account, the models developed can be considered good for all the minerals analyzed and all the equations developed fulfilling this criterion.

In order to check the validity of the predictive models developed, an external validation was carried out. For this purpose, 33 samples (20% of the samples analyzed) which had not participated in the development of the models were analyzed. For this purpose, the calibration equations obtained were applied to the spectra of the samples reserved for external validation. The suitability of the model developed was assessed by comparing both results through the Student *t*-statistic and the RMSE error. Table 5 shows the results of the Student’s *t*-test for paired values when comparing the reference data (obtained in the laboratory by ICP-MS) and the values obtained from the NIRS equations for these 33 samples. The p-values were higher than 0.05 for all the minerals evaluated, which means that there are no significant differences between the values provided by the two methods. The RMSE (root mean standard error) obtained ranged from 0.006 for Mg to 8.590 for Fe. The high RMSE value obtained in the prediction of Fe indicates a lower accuracy in the determination of Fe content. In spite of this, the results obtained show that the prediction equations were satisfactory for determining the mineral content of lentil flours and flours fortified with lentil flour.

## 4. Conclusions

NIR technology has proven to be an effective tool for establishing the presence of lentil flour in fortified samples and for the quantification of its mineral content. Flours could be satisfactorily discriminated according to the percentage of lentil present in them. Furthermore, promising results were obtained in the discrimination of the lentil variety used. Regarding the mineral composition, the models developed have proved to be suitable for the determination of the Ca, Mg, Fe, P and K content of the flours. The range of applicability of the calibration equations obtained by NIR with a fiber optic probe is comparable to that obtained by the reference technique (ICP_MS). The multiparametric character of NIR technology combined with its low cost and rapid analysis make it a very promising tool in the field of vegetable flours and their formulations.

## Figures and Tables

**Table 1:** Composition and number of flour samples analyzed.

Sample	Composition	Lentil Variety	Nº of Samples	Total Samples
Commercial wheat flour	100% wheat	-	3	3
Lentil flour	100% lentil	Castellana	20	60
		Pardina	20	
		Guareña	20	
Fortified flour	75% wheat/25% lentil	Castellana	10	90
		Pardina	10	
		Guareña	10	
	50% wheat/50% lentil	Castellana	10	
		Pardina	10	
		Guareña	10	
	25% wheat/75% lentil	Castellana	10	
		Pardina	10	
		Guareña	10	

**Table 2:** Results of the classification of the flours according to the percentage of lentil flour present, based on the mathematical pre-treatments applied to the NIR spectra that provided the best results.

		Global Composition Results		Validation Results by Category				Global Lentil Variety Results		Validation Results by Category		
		Correctly Classified (%)		Sensitivity				Correctly Classified (%)		Sensitivity		
Spectral Pre-Treatment		Calibration (n = 120)	Validation (n = 33)	100	75	50	25	Calibration (n = 120)	Validation (n = 33)	Castellana	Guareña	Pardina
None	0-0-1-1	98.06	95.56	0.88	1.00	1.00	1.00	77.50	70.00	0.80	0.80	0.50
	1-4-4-1	100.00	100.00	1.00	1.00	1.00	1.00	82.50	76.67	0.70	0.60	1.00
	2-4-4-1	100.00	100.00	1.00	1.00	1.00	1.00	94.17	93.33	1.00	0.80	1.00
	2-10-10-1	100.00	100.00	1.00	1.00	1.00	1.00	83.33	76.67	0.70	0.60	1.00
	2-8-6-1	100.00	100.00	1.00	1.00	1.00	1.00	84.17	80.00	0.70	0.70	1.00
Detrend	0-0-1-1	98.88	95.55	0.88	1.00	1.00	1.00	77.50	73.33	0.80	0.50	0.90
	1-4-4-1	100.00	100.00	1.00	1.00	1.00	1.00	84.17	76.67	0.70	0.60	1.00
	2-4-4-1	100.00	100.00	1.00	1.00	1.00	1.00	94.17	93.33	1.00	0.80	1.00
	2-10-10-1	100.00	100.00	1.00	1.00	1.00	1.00	83.33	76.67	0.70	0.60	1.00
	2-8-6-1	100.00	100.00	1.00	1.00	1.00	1.00	84.17	80.00	0.70	0.70	1.00
SNV	0-0-1-1	96.94	93.33	0.83	1.00	1.00	1.00	66.67	63.33	0.60	0.40	0.90
	1-4-4-1	100.00	100.00	1.00	1.00	1.00	1.00	88.33	86.67	0.70	0.90	1.00
	2-4-4-1	100.00	100.00	1.00	1.00	1.00	1.00	100.00	96.67	1.00	0.90	1.00
	2-10-10-1	100.00	98.88	1.00	0.94	1.00	1.00	88.33	73.33	0.80	0.40	1.00
	2-8-6-1	100.00	100.00	1.00	1.00	1.00	1.00	90.00	83.33	0.80	0.90	0.80
SNV-Detrend	0-0-1-1	100.00	100.00	1.00	1.00	1.00	1.00	87.5	80.00	0.80	0.60	1.00
	1-4-4-1	100.00	100.00	1.00	1.00	1.00	1.00	93.33	80.00	0.70	0.70	1.00
	2-4-4-1	100.00	100.00	1.00	1.00	1.00	1.00	100.00	96.67	1.00	0.90	1.00
	2-10-10-1	100.00	98.88	1.00	0.94	1.00	1.00	88.33	73.33	0.80	0.40	1.00
	2-8-6-1	100.00	100.00	1.00	1.00	1.00	1.00	90.00	83.33	0.80	0.90	0.80

100: flours with a percentage of 100% lentil flour; 75: flours with 75% lentil flour/25% wheat flour; 50: flours with 50% lentil flour/50% wheat flour; 25: flours with 25% lentil flour/75% wheat flour.

**Table 3:** Mineral composition of the lentil and wheat flours analyzed; maximum and minimum values, mean value and standard deviation (SD).

	K (g/100 g)		P (g/100 g)		Mg (g/100 g)		Ca (g/100 g)		Fe (mg/kg)	
	Min-Max	$\bar{x}\pm SD$	Min-Max	$\bar{x}\pm SD$	Min-Max	$\bar{x}\pm SD$	Min-Max	$\bar{x}\pm SD$	Min-Max	$\bar{x}\pm SD$
Lentil flour										
var. Castellana,	0.78–0.94	0.87 ± 0.05 ef	0.27–0.40	0.34 ± 0.04 e	0.09–0.11	0.09 ± 0.01 e	0.08–0.12	0.10 ± 0.01 f	45.60–75.00	61.62 ± 10.57 de
var. Guareña	0.90–1.02	0.94 ± 0.04 f	0.37–0.45	0.41 ± 0.02 f	0.09–0.11	0.09 ± 0.01 e	0.07–0.10	0.08 ± 0.01 e	42.30–106.00	67.65 ± 15.65 de
var. Pardina	0.79–0.99	0.81 ± 0.26 de	0.33–0.44	0.39 ± 0.04 f	0.09–0.11	0.10 ± 0.06 e	0.07–0.10	0.08 ± 0.01 de	44.70–115.00	73.24 ± 24.21 e
Wheat flour										
Non fortified	0.14–0.17	0.16 ± 0.01 a	0.01–0.92	0.09 ± 0.26 a	0.02–0.30	0.02 ± 0.11 a	0.02–0.29	0.02 ± 0.12 a	10.56–14.60	11.97 ± 1.27 a
25% Fortified	0.14–0.37	0.34 ± 0.04 b	0.14–0.18	0.16 ± 0.01 b	0.04–0.05	0.04 ± 0.01 b	0.03–0.05	0.04 ± 0.00 b	20.12–38.30	26.42 ± 4.50 b
50% Fortified	0.20–0.60	0.51 ± 0.08 c	0.18–0.28	0.24 ± 0.02 c	0.06–0.07	0.06 ± 0.00 c	0.05–0.07	0.05 ± 0.01 c	27.52–63.87	40.12 ± 9.00 c
75% Fortified	0.40–0.80	0.69 ± 0.12 d	0.23–0.38	0.31 ± 0.03 d	0.07–0.09	0.08 ± 0.01 d	0.06–0.10	0.07 ± 0.01 d	34.91–89.43	53.81 ± 13.51 d

a–f: different letters in the same column indicate statistically significant differences; analysis of variance (ANOVA); post hoc: Tukey HSD test; *p* < 0.05.

**Table 4:** Statistical descriptors for the best calibration equations obtained from NIR spectra and mineral composition data obtained by ICP-MS on flour samples.

Mineral	Math Treatment	N	Mean	SD	Min Est.	Max Est.	SEC	SECV	RSQ
Mg	SNV 2,4,4,1	114	0.0767	0.0205	0.0153	0.1381	0.0038	0.0048	0.96
P	Detrend 2,10,10,1	110	0.292	0.0911	0.0187	0.5653	0.0206	0.0208	0.94
K	Detrend 0,0,1,1	115	0.6794	0.2211	0.0162	1.3426	0.0293	0.039	0.98
Ca	Detrend 0,0,1,1	115	0.0685	0.0211	0.0052	0.1316	0.0051	0.0056	0.94
Fe	SNV 2,4,4,1	108	49.1007	18.2117	0.0000	103.7358	6.5112	8.984	0.87

N: number of samples after removing the outliers; SNV; standard normal variate; SD: standard deviation; RSQ: multiple correlation coefficients; SEC: standard error of calibration; SECV: standard error of cross-validation.

**Table 5:** External validation for the NIR determination of minerals in fortified flours.

Mineral	*p* (Level of Significance)	RMSE *
Mg	0.83	0.006
P	0.13	0.014
K	0.38	0.034
Ca	0.85	0.008
Fe	0.67	8.590

* RMSE: root mean standard error.

## Data Availability

The data presented in this study are available on request from the corresponding author.
